# Royal United Hospital, Bath.

**Published:** 1905-07-01

**Authors:** 


### HOSPITAL ADMINISTRATION.

### CONSTRUCTION AND ECONOMICS.

### ROYAL UNITED HOSPITAL, BATH.

At the unanimous request of the Board of this institution Sir Henry Burdett undertook the responsible duty of inspecting the hospital and its administration, the examination of its accounts, and the consideration of the privileges of its subscribers. He found the financial position to be serious, seeing that the overdraft at the bank was about £5,000 and the annual expenses exceeded the income from subscriptions and donations by some £1,800 per annum. Sir Henry reports as the result of his examination that he does not find any indication of unnecessary expenditure in any department. An inspection of the buildings showed the wards especially to be deficient in many things, as much of the furniture is very old and should be renewed. Sir Henry also found that a nurses' home is essential, and he reports further that the time is near when the Governors should seriously contemplate the pulling down of most of the present buildings and the erection of a modern hospital by preference on a more suitable and less restricted site. After reporting fully on the matters referred to him and showing the causes which underlie the present condition of affairs, Sir Henry recommends:—

1. That the Royal United Hospital should be turned at once into a free institution, and that subscriptions should be invited without privileges on the basis of pure charity; or, that the present privileges of subscribers should be modified by raising the qualification for a governor to two guineas annual subscription, or £25 in one sum; each subscriber of two guineas to be entitled to one in-patient letter or to 12 out-patient letters, and smaller subscribers to be entitled to out-patient letters on the following scale: one out-patient letter for an annual subscription of 5s. a year, three out-patient letters for 10s. 6d. a year, six out-patient letters for 21s. a year.

2. That £5,000, being the amount due to the Treasurer, on December 31st, 1904, or so much as may remain undischarged, shall be repaid by realising a sufficient amount of the investments to discharge this debt.

3. That the uniform system of accounts be introduced without delay.

4. That steps be taken to increase the area of support by organising the districts from which patients are sent on a plan which shall divide it into towns and villages, and set out the actual amount of subscriptions received from each such town or village, and the annual value of the relief afforded to the inhabitants thereof as represented by the cost of such relief at the Royal United Hospital.

5. That the annual subscriptions shall become due on a given day, and that no tickets shall be issued in any year until the subscriptions for that year have been received.

The report is dated May 9, 1905, and after fully considering it the Committee summoned a general meeting of the subscribers, to deal with the matter, which was held in Bath on June 21. The Committee recommended the governors to adopt recommendation I., *i.e.* the free system, but, after considerable discussion, on the motion of Prebendary N. Thompson, the matter was adjourned until the autumn to allow time for the full consideration of Sir Henry Burdett's report and all the issues which underlie it.

Speaking at a public meeting at Bath held last week Sir Henry Burdett pointed out that something must be done or the institution would soon be bankrupt. He explained that the ticket system in modern days did not in practice confer upon the governors a definite right to admission, as all cases applying for treatment to a hospital had to be considered from the point of view of their bodily ailments and needs. Whether the subscribers determined to abolish the ticket system, or to modify the present plan of issuing tickets, the responsibility to provide a considerably larger income must in either case rest on the shoulders of the inhabitants of Bath and the district within a radius of 21 miles of that city. If the spirit of charity were warm enough and strong enough to ensure that by the free system the necessary funds could without doubt be procured, then it would be desirable to adopt it. If, on the other hand, the free system were merely an ideal in the minds of certain enthusiastic supporters of the hospital, and the only way, as many of the speakers maintained, to secure funds was to continue a system of tickets, it would be far better for practical purposes to give up the ideal for the moment at any rate, because the work of the hospital must be carried on. The following resolution, which was supported by the vicar and several representatives of the working classes, was passed: "That this meeting having heard the result of Sir Henry Burdett's investigations into the affairs of the Royal United Hospital desires to record its confidence in the management of the institution and pledges itself to do its utmost to secure the increased financial support so urgently needed." Mr. Egbert Lewis, the chairman of the Board, proposed a vote of thanks to Sir Henry Burdett for his report, which was seconded by Sir John Goldney and supported by the Mayor, who stated that although they were only considering but one phase of the report that day they did not forget the other recommendations, which would be dealt with in due course.

